# Persistent Firing in Hippocampal CA1 Pyramidal Cells in Young and Aged Rats

**DOI:** 10.1523/ENEURO.0479-22.2023

**Published:** 2023-03-28

**Authors:** Yacine Brahimi, Beate Knauer, Alan Tobias Price, Maria Jesus Valero-Aracama, Antonio Reboreda, Magdalena Sauvage, Motoharu Yoshida

**Affiliations:** 1 German Center for Neurodegenerative Diseases (DZNE), Magdeburg 39120, Germany; 2 Otto‐von‐Guericke Universität (OvGU), Medical Faculty, Magdeburg 39120, Germany; 3 International Graduate School of Neuroscience (IGSN), Ruhr University Bochum (RUB), Bochum 44801, Germany; 4 Leibniz Institute for Neurobiology (LIN), FAM Dpt, Magdeburg 39118, Germany; 5 Center for Behavioral Brain Sciences (CBBS), Magdeburg 39106, Germany; 6 Faculty of Psychology, Mercator Research Group-Structure of Memory, Ruhr University Bochum, Bochum 44801, Germany

**Keywords:** afterhyperpolarization, aged rats, cholinergic agonist, depolarization current, hippocampus, persistent firing

## Abstract

Persistent neuronal firing is often observed in working memory and temporal association tasks both in humans and animals, and is believed to retain necessary information in these tasks. We have reported that hippocampal CA1 pyramidal cells are able to support persistent firing through intrinsic mechanisms in the presence of cholinergic agonists. However, it still remains largely unknown how persistent firing is affected by the development of animals and aging. Using *in vitro* patch-clamp recordings from CA1 pyramidal cells in rat brain slices, we first show that the cellular excitability of these aged rats was significantly lower than that of the young rats, responding with fewer spikes to current injection. In addition, we found age-dependent modulations of input resistance, membrane capacitance, and spike width. However, persistent firing in aged (approximately two-year-old) rats was as strong as that in young animals, and the properties of persistent firing were very similar among different age groups. In addition, medium spike afterhyperpolarization potential (mAHP), was not increased by aging and did not correlate with the strength of persistent firing. Lastly, we estimated the depolarization current induced by the cholinergic activation. This current was proportional to the increased membrane capacitance of the aged group and was inversely correlated with their intrinsic excitability. These observations indicate that robust persistent firing can be maintained in aged rats despite reduced excitability, because of the increased amount of cholinergically induced positive current.

## Significance Statement

In an aging society, it is crucial to understand neural mechanisms underlying age-dependent cognitive impairments. In recent years, the importance of intrinsic cellular properties in cognitive functions such as memory has increasingly been recognized. However, research examining age-dependent alteration of intrinsic cellular mechanisms of persistent firing, which is believed to support working memory function, has so far been very scarce. In this study, we demonstrate that the ability to support persistent firing is kept intact in neurons from old rats, despite changes in other properties such as intrinsic excitability. These results identify the ability to support persistent firing as one potential cellular mechanism of rescued cognitive functions under cholinergic enhancement used to treat Alzheimer’s disease and other dementias.

## Introduction

Working memory is the ability to maintain necessary information for a short period of time (up to tens of seconds) and is a crucial cognitive function that we use in daily life (Miller et al., 2018). Working memory declines in aged human subjects and animals (Gazzaley et al., 2005; Bizon et al., 2012). However, the cellular mechanism underlying working memory and its decline in aging remain largely unknown (Wang et al., 2011).

Persistent firing, which is a repetition of action potentials that outlasts the triggering stimulus, is believed to be a cellular correlate of working memory (Major and Tank, 2004; Reboreda et al., 2011; Zylberberg and Strowbridge, 2017; Oh and Disterhoft, 2020). Persistent firing is often observed during the maintenance phase of working memory and temporal association tasks when short-term retention of information is required (Major and Tank, 2004). Persistent firing during working memory has been shown to be task performance-relevant (Goldman-Rakic, 1995; Wang et al., 2007) and is altered in aged animals (Wang et al., 2011).

*In vitro* studies have shown that persistent firing can be supported by intrinsic cellular mechanisms within individual neurons in many brain areas associated with working memory and temporal association tasks (Reboreda et al., 2018). This intrinsic persistent firing can be supported by cholinergic activation (Klink and Alonso, 1997; Egorov et al., 2002; Knauer et al., 2013) which is crucial for working memory performance (Hasselmo, 2006), and the neuromodulatory effects on intrinsic persistent firing are similar to those on working memory performance and persistent firing observed *in vivo* (Zhang et al., 2013; Reboreda et al., 2018; Valero-Aracama et al., 2021). Furthermore, a correlation between such persistent firing recorded *in vitro* and temporal association behavior has recently been reported (Lin et al., 2020).

As for the mechanism underlying this type of persistent firing, the roles of neuromodulators such as acetylcholine and noradrenaline, and the involvement of molecular mechanisms such as TRPC channels, have been characterized intensively by others and us (Yan et al., 2009; Zhang et al., 2011, 2013; Knauer and Yoshida, 2019; Arboit et al., 2020). The involvement of modulatory pathways such as the cholinergic system and the PKA pathway suggests that there may be age-dependent changes in persistent firing. However, data on how development and aging affect this intrinsic persistent firing remains scarce (Reboreda et al., 2007; Lin et al., 2020). Thus far, there is no available data on the effect of development and aging on intrinsic persistent firing in hippocampal neurons, leaving it unknown whether persistent firing is affected by these factors.

Here, we compared intrinsic persistent firing in young rats during development (2–3.5 weeks postnatal) with aged rats (>87 weeks) in hippocampal CA1 pyramidal cells using *in vitro* patch-clamp recordings. We first compared intrinsic excitability and membrane properties that are known to be involved in persistent firing and modulated by aging. We find that intrinsic excitability is significantly reduced in the aged group as reported by other groups (Disterhoft and Oh, 2007; Oh et al., 2010, 2016), indicating that aging did affect cellular properties of the neurons we used in this study. However, to our surprise, persistent firing is as strong in the aged group as the young groups. In addition, we demonstrate that medium spike afterhyperpolarization potential (mAHP), which is known to modulate persistent firing in other brain areas, did not correlate with the strength of persistent firing. We then demonstrate that the depolarization current induced by the cholinergic activation was proportional to the larger membrane capacitance of the aged neurons, and was inversely correlated with their intrinsic excitability. In summary, our results suggest that robust intrinsic persistent firing can be maintained in aged animals despite significant reduction of intrinsic excitability, potentially because of an increased cholinergically induced inward current. We further discuss the relevance of these findings to age-dependent working memory decline and degeneration of the cholinergic system. Some of these results have been presented in the form of a thesis (Knauer, 2016).

## Materials and Methods

All experiments involving animals were conducted at the Ruhr University Bochum, Bochum, Germany, in 2011–2014, in accordance with the guidelines of the local animal ethics committee and with the European Communities Council Directive of September 22, 2010 (2010/63/EU). Furthermore, the principles of laboratory animal care and use were followed (National Research Council, 2011).

### Animals

Long–Evans rats of either sex used in this study were obtained from Charles River and Janvier parental stock. While the young (postnatal day (P) 14–24) groups of rats were all naive animals, the aged group animals (24–27 months), had diverse histories because we used existing available animals in accordance with the reduction criterion in the 3R principle (Tannenbaum and Bennett, 2015). The majority (four out of six rats) of the aged group rats went through training in a water maze task. They were not tested for their performance and their performance during the training was not noted. The rest (two out of six rats) did not go through this training. The period between the last day of training and slicing was between 19 and 27 d. This behavioral training was not part of our experimental design. We report it here solely for the purpose of describing the nature of the aged rats used in our study based on previous publications reporting the effect of behavioral training on cellular excitability in a different strain of rat (Disterhoft and Oh, 2007; Kuo et al., 2008; Oh et al., 2010) and persistent firing in a different strain and type of cell (Lin et al., 2020). All aged rats were male. Cells from the juvenile groups were recorded from both male and female rats. The P14–P15 group was composed of three females (57% of n_P14–15_ cells) and three males. The P16–P19 group was composed of five females (70% of n_P16–19_ cells) and three males, while the P20–P24 group was composed of six females (75% of n_P20–24_ cells) and two males. All animals were group housed.

### Preparation of acute brain slices

Upon loss of the pedal reflex under ketamine:xylazine anesthesia (100:4 mg/kg), animals were transcardially perfused with ice-cold cutting solution containing (in mm) 110 choline chloride, 1.25 NaH_2_PO_4_, 7 MgCl_2_, 2.5 KCl, 7 D-glucose, 3 pyruvic acid, 1 ascorbic acid, 26 NaHCO_3_, and 0.5 CaCl_2_. After removal of the brain from the cranial cavity, slices were cut horizontally at a thickness of 350 μm in ice-cold cutting solution using a vibratome (VT1000 S, Leica Instruments). Immediately after each cut, slices were individually transferred to 30°C artificial CSF (ACSF) containing (in mm) 124 NaCl, 1.25 NaH_2_PO_4_, 1.8 MgSO_4_, 3 KCl, 10 D-glucose, 26 NaHCO_3_, and 1.6 CaCl_2_ and remained there for ∼30 min. Subsequently, slices were kept at room temperature for at least 30 min, then maintained at room temperature until recording commenced. All solutions were continuously aerated and pH adjusted with 95% O_2_ and 5% CO_2_ (carbogen). These protocols were previously described in more detail (Knauer, 2016; Knauer and Yoshida, 2019).

### Recording procedures

Brain slices were submerged onto a nylon mesh in continuously flowing (2 ml/min), 35 ± 1°C, carbogen aerated ACSF, additionally containing kynurenic acid (2 mm) and picrotoxin (0.1 mm) to block fast ionotropic glutamate and GABA_A_ synaptic transmissions, respectively.

Whole-cell recordings from neurons in the CA1 stratum pyramidale were conducted using freshly pulled borosilicate glass pipettes (3–8 MΩ) filled with intracellular fluid containing (in mm) 120 K-gluconate, 10 HEPES, 0.2 EGTA, 20 KCl, 2 MgCl_2_, 7 PhCreat di(tris), 4 Na_2_-ATP, 0.3 GTP tris salt, and 0.1% biocytin (pH adjusted to 7.3 using KOH). A liquid junction potential of ∼10 mV was not corrected. Cell signals were sampled at 20 kHz in the current-clamp mode, amplified with an Axo-Clamp-2A amplifier (Molecular Devices), low pass filtered at 10 kHz, and recorded with Clampex 9.0 data acquisition software. These protocols were previously described in more detail (Knauer, 2016; Knauer and Yoshida, 2019).

### Chemicals and solutions

Chemicals were purchased from Sigma-Aldrich (ascorbic acid, biocytin, choline chloride, potassium D-gluconate, Na_2_-ATP, phosphocreatine di(tris), pyruvic acid, tris-GTP, and kynurenic acid), J.T. Baker (CaCl_2_, KOH, NaCl, NaHCO_3_, NaH_2_PO_4_), TCI Europe (picrotoxin), AppliChem (glucose), Roth (MgCl_2_), and VWR (KCl). Carbamoylcholine chloride (carbachol or Cch) was obtained from Alfa Aesar and dissolved in distilled water to obtain a 10 mm stock solution.

### Identification of anatomic locations of cells

To track the slice identity (left or right and serial position) and obtain metric measures, the approximate dorsoventral position of recorded cells was determined based on the slice number, whether cells were recorded from the dorsal or ventral face of the slice, and the height of the remaining brain. The cells’ proximo-distal location within CA1 was determined *post hoc* from microscope pictures after horseradish-peroxidase based staining of biocytin (DAB kit, SK-4100, Vector Laboratories). The proximo-distal location was quantified as the ratio of the distance of the cell from the CA1–CA2 border over the total length of CA1 by a rater blind to the electrophysiological properties of the cells. The measurements were made following the curvature of the stratum pyramidale. These protocols were previously described in more detail (Knauer, 2016; Knauer and Yoshida, 2019).

Cells from aged animals happened to be recorded exclusively in the distal half of CA1 (52–96%). In order to assure that any differences observed between young and aged animals were not because of this sampling bias, only cells in the distal half of CA1 (51–93%) were accepted for data analysis of the young group.

### Data analysis

Cells spontaneously firing action potentials after membrane break-in were excluded from data analysis. Additionally, cells from which regular action potentials were not overshooting 0 mV were excluded from data analysis. Recordings were analyzed using custom-written codes in Python 3.

Intrinsic properties of cells held at −70 mV were assessed by applying square pulses of 1 s with current amplitudes varying from −300 to 400 pA in increments of 50 pA. Hereafter, this protocol is referred to as the current-voltage (I–V) protocol. Spike properties were measured from the first traces that generated at least 15 action potentials. The input resistance was obtained from Ohm’s law by dividing the membrane potential deflection ΔV_membrane_ by the amplitude of the injected current step (−50 pA). The membrane time constant was estimated from a single exponential fit of voltage responses following a current step of −50 pA. Capacitance was estimated from the ratio of the membrane time constant over the input resistance. The sag ratio was assessed in response to a −300-pA direct current injection and defined as the fraction of the sag amplitude and the sag peak deflection from the baseline. To control for differences in input resistance, the negative peak amplitude was normalized to the baseline.

The action potential threshold was measured as the membrane potential at which the third-order derivative of the membrane potential was maximum. The spike amplitude was measured as the voltage difference between the action potential threshold and the maximum of the action potential. The average full width at half maximum and full width at one-third maximum was calculated as the time between the point at which the membrane potential reaches half or one-third of the spike amplitude, respectively. The maximum somatic depolarization rate (dV/dt) preceding the action potential peak was calculated as the maximum value of the first-order derivative of the membrane potential between the action potential threshold and action potential peak. The maximum somatic repolarization rate (dV/dt) following the action potential peak was calculated as the minimum value of the first-order derivative of the membrane potential between the action potential peak and 3 ms following the peak. The adaptation ratio was measured as the ratio of the instantaneous frequency of the last and last-but-one action potentials to the instantaneous frequency of the second and third action potentials. The fast doublet index was calculated as the ratio of the instantaneous frequency of the first and second action potentials to the instantaneous frequency of the second and third action potentials.

The approximate firing threshold of individual cells was experimentally determined by manual adjustments to the injected current. Persistent firing induction was then evaluated by bringing the membrane potential just below the spike threshold. From there, a 100-pA square pulse of 2-s duration was applied and the poststimulus responses were monitored for 45 s. Recordings were first visually inspected and classified as persistent firing (PF), depolarization block (DB), or none (No PF) based on their responses after the stimulus offset. Cells were defined as having persistent firing when they fired action potentials for at least 10 s after the cessation of the stimulation pulse. When persistent firing lasted for >30 s, cells were classified as having long-lasting persistent firing. Cells were classified as DB when the membrane potential depolarized above the firing threshold for >5 s continuously after the cessation of the stimulus.

The baseline was measured as the average membrane potential 200 ms before each stimulus in all protocols. The medium AHP (mAHP) amplitude was measured as the difference between the mAHP peak and the baseline both from the I-V protocol (50–400 pA, 1 s) and persistent firing induction protocol (100 pA, 2 s). The slow AHP (sAHP) was induced by the same protocols and was measured as the membrane potential value at 1 s after the stimulus offset. Interspike intervals (ISIs) were measured as the duration of time between the peaks of action potentials. The coefficient of variation (CV) of ISI was calculated as the ratio of the ISI standard deviation to the ISI mean.

### Statistical analysis

Normality was assessed using Shapiro–Wilk and D’Agostino–Pearson tests. Variance homogeneity was checked with Brown–Forsythe and Barlett’s tests. Whenever both normality and variance were verified, one-way ANOVA or repeated measures (RM) two-way ANOVA analyses were performed depending on the data structure. When the assumption of sphericity was not verified for RM two-way ANOVA, the Greenhouse–Geisser correction was applied. When variance homogeneity was not verified, Welch’s one-way ANOVA was performed. Whenever the normality assumption was not verified, nonparametric Kruskal–Wallis and Mann–Whitney tests were performed; *p*-values from multiple pairwise comparisons were corrected with Tukey’s honestly significant difference (HSD) test for parametric tests when the homogeneity of the variances was verified. Otherwise, Tamhane’s T2 tests were performed. For nonparametric tests, Dunn’s multiple comparison correction was applied. Generalized linear mixed-model analysis (γ probability distribution and log link function) was used for non-normal factorial repeated measures designs. Significance level ɑ, 0.05 (*p < 0.05, **p < 0.01, ***p < 0.001, ****p < 0.0001) was used. Boxplot’s center lines represent the median, the box length is defined by the 25th and 75th percentiles, and whiskers represent the highest and lowest data point, excluding outliers.

## Results

### Comparison of intrinsic excitability

First, we asked whether membrane properties and intrinsic excitability were different among the young and aged groups. The young group consisted of rats in the postnatal days (P) 14 to 24 during which the cholinergic system, which is critically involved in working memory and temporal bridging tasks (Hasselmo, 2006), develops and reaches maturation (Gould et al., 1991; Kiss and Patel, 1992; Zahalka et al., 1993). The young group was further subdivided into three age groups: P14–P15 (*n*_cells_ = 7), P16–P19  (*n*_cells_ = 10), and P20–P24 (*n*_cells_ = 8). This age binning was selected to refine the observation of electrophysiological properties during critical postnatal time periods for cholinergic system development. The aged group rats consisted of six two-year-old (24–27 month old) rats (*n*_cells_ = 10). Three cells from the two rats that were not assigned to the behavioral project (see Materials and Methods) are marked with black triangles in the following figures for clarity. This behavioral training was not part of our experimental design.

**Figure 1.**
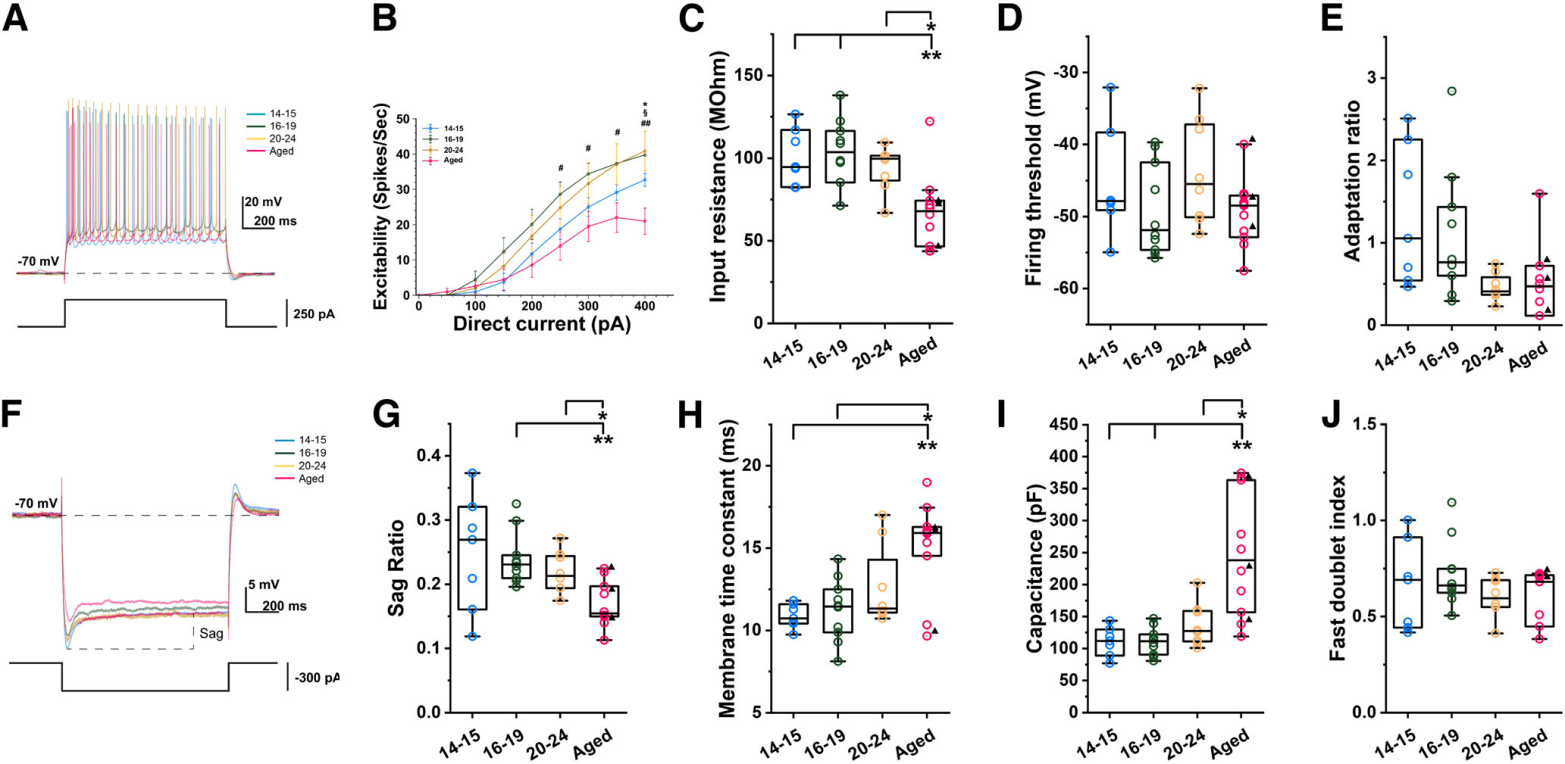
Membrane properties of CA1 pyramidal cells in young and aged rats in ACSF. ***A***, Example voltage traces used to calculate intrinsic excitability for all age groups. Bottom trace shows injected current (1 s, 250 pA). ***B***, Intrinsic excitability measured as number of action potentials elicited during the 1-s current injection. * represents a significant difference between P14–P15 and Aged (Tukey’s *post hoc* test, *p* < 0.05); # represents a significant difference between P16–P19 and Aged (Tukey’s *post hoc* test, *p* < 0.05); § represents a significant difference between P20–P24 and Aged (Tukey’s *post hoc* test, *p* < 0.05). ***C***, Input resistance (Tukey’s *post hoc* test, P14–P15 vs Aged, *p* = 0.0073; P16–P19 vs Aged, *p* = 0.0013; P20–P24 vs Aged, *p* = 0.0349). ***D***, Spike threshold. ***E***, Adaptation ratio. ***F***, Example voltage traces used to calculate sag ratio. Bottom trace shows injected current (1 s, −300 pA). ***G***, Sag ratio (Tamhane’s T2 *post hoc* test, P16–P19 vs Aged, *p* = 0.0042; P20–P24 vs Aged, *p* = 0.0432). ***H***, Membrane time constant (Tamhane’s T2 *post hoc* test, P14–P15 vs Aged, *p* = 0.0078; P16–P19 vs Aged, *p* = 0.0203). ***I***, Membrane capacitance (Tamhane’s T2 *post hoc* test, P14–P15 vs Aged, *p* = 0.0095; P16–P19 vs Aged, *p* = 0.0098; P20–P24 vs Aged, *p* = 0.0392). ***J***, Fast doublet index (one-way ANOVA, *p* = 0.3722, *F*_(3,28)_ = 1.083).

In the absence of cholinergic agonist, carbachol, we first measured intrinsic excitability by counting the number of elicited spikes during a brief (1 s) current injection with various amplitudes (50–400 pA; Fig. 1*A*). We found that intrinsic excitability was smaller in the aged group than in the young groups in general (RM two-way ANOVA, *p* = 1.779e-5; Fig. 1*B*). The input resistance of the aged group was smaller compared with the young groups (one-way ANOVA, *p* = 0.001, *F*_(3,31)_ = 6.94; Fig. 1*C*), while the firing threshold was not significantly different (one-way ANOVA, *p* = 0.2318, *F*_(3,31)_ = 1.51; Fig. 1*D*). In addition, adaptation ratio tended to decrease with aging (Welch’s one-way ANOVA, *p* = 0.0371, *F*_(3,14.39)_ = 3.69; Fig. 1*E*). As described more in the discussion section, a larger input resistance in the young groups is in line with earlier literature (Vasilyev and Barish, 2002; Reboreda et al., 2007). These data indicate that the reduced intrinsic excitability could be because of the lower input resistance of the aged group.

Second, we tested the sag ratio, which is indicative of the presence of the h-current, because the involvement of the h-current in persistent firing has been reported (Matos et al., 2015). The sag ratio of the aged group was smaller than that in the P16 to P19 and P20 to P24 groups (Welch’s one-way ANOVA, *p* = 0.0058, *F*_(3,15.53)_ = 6.14; Fig. 1*F*,*G*). Finally, the membrane time constant in the aged group was longer than that in the P14 to P15 and P16 to P19 groups (Welch’s one-way ANOVA, *p* = 0.0043, *F*_(3,15.92)_ = 6.54; Fig. 1*H*), and the membrane capacitance of the old group was higher than any of the young groups (Welch’s one-way ANOVA, *p* = 0.004, *F*_(3,15.99)_ = 6.66; Fig. 1*I*). This is in line with previously published data indicating increased membrane capacitance with development (Vasilyev and Barish, 2002).

**Figure 2.**
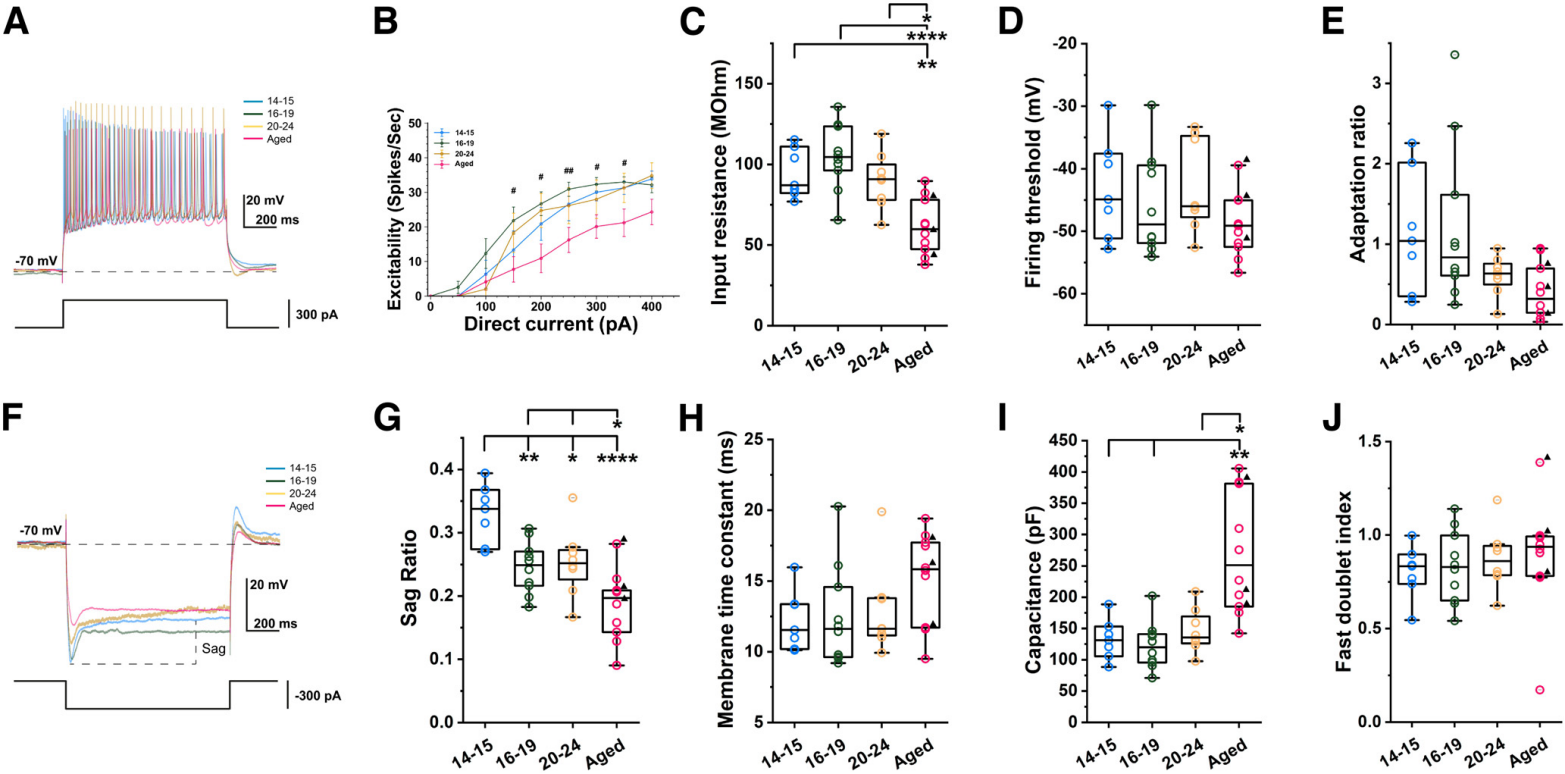
Membrane properties of CA1 pyramidal cells in young and aged rats with carbachol. ***A***, Example voltage traces used to calculate intrinsic excitability for all age groups. Bottom trace shows injected current (1 s, 300 pA). ***B***, Intrinsic excitability measured as numbers of action potential elicited during the 1-s current injection. # represents a significant difference between P16–P19 and Aged (Tukey’s *post hoc* test, *p* < 0.05). ***C***, Input resistance (Tukey’s *post hoc* test, P14–P15 vs Aged, *p* = 0.0039; P16–P19 vs Aged, *p* = 3.93e-5; P20–P24 vs Aged, *p* = 0.0108). ***D***, Spike threshold. ***E***, Adaptation ratio. ***F***, Example voltage traces used to calculate sag ratio. Bottom trace shows injected current (1 s, −300 pA). ***G***, Sag ratio (Tukey’s *post hoc* test, P14–P15 vs P16–P19, *p* = 0.0083; P14–P15 vs P20–P24, *p* = 0.0258; P14–P15 vs Aged, *p* = 7.78e-6; P16–P19 vs Aged, *p* = 0.0446; P20–P24 vs Aged, *p* = 0.0307). ***H***, Membrane time constant. ***I***, Membrane capacitance (Tamhane’s T2 *post hoc* test, P14–P15 vs Aged, *p* = 0.0087; P16–P19 vs Aged, *p* = 0.0049; P20–P24 vs Aged, *p* = 0.0177). ***J***, Fast doublet index (one-way ANOVA, *p* = 0.8535, *F*_(3,31)_ = 0.260).

Because persistent firing was tested in the presence of carbachol, a nonselective cholinergic agonist, we next examined the same membrane properties also in the presence of carbachol in the same sets of cells (Fig. 2). The intrinsic excitability was again the smallest in the aged group (RM two-way ANOVA, *p* = 0.0230, *F*_(21,211)_ = 1.77; Fig. 2*B*) and the difference was as large or even larger when compared with the condition without carbachol (Fig. 2*A*,*B*). Similarly to the condition without carbachol, the measurements of the input resistance and firing threshold indicate that this lower intrinsic excitability may arise from the lower input resistance (one-way ANOVA, *p* = 6.305e-5, *F*_(3,31)_ = 10.51; one-way ANOVA, *p* = 0.2622 *F*_(3,31)_ = 1.40, respectively; Fig. 2*C*,*D*). The sag ratio of the aged group was smaller than any of the three young groups (one-way ANOVA, *p* = 2.331e-5, *F*_(3,31)_ = 11.93; Fig. 2*F*,*G*), the membrane time constant of the aged group was not significantly higher than those of the young groups in the presence of carbachol (Welch’s one-way ANOVA, *p* = 0.1784, *F*_(3,16.99)_ = 1.84; Fig. 2*H*), and the membrane capacitance of the old group was again higher than any of the young groups (Welch’s one-way ANOVA, *p* = 0.0044, *F*_(3,16.78)_ = 6.38; Fig. 2*I*). In summary, these analyses indicate that membrane properties were different between the young and aged groups regardless of the presence of carbachol.

### Spike properties

**Figure 3.**
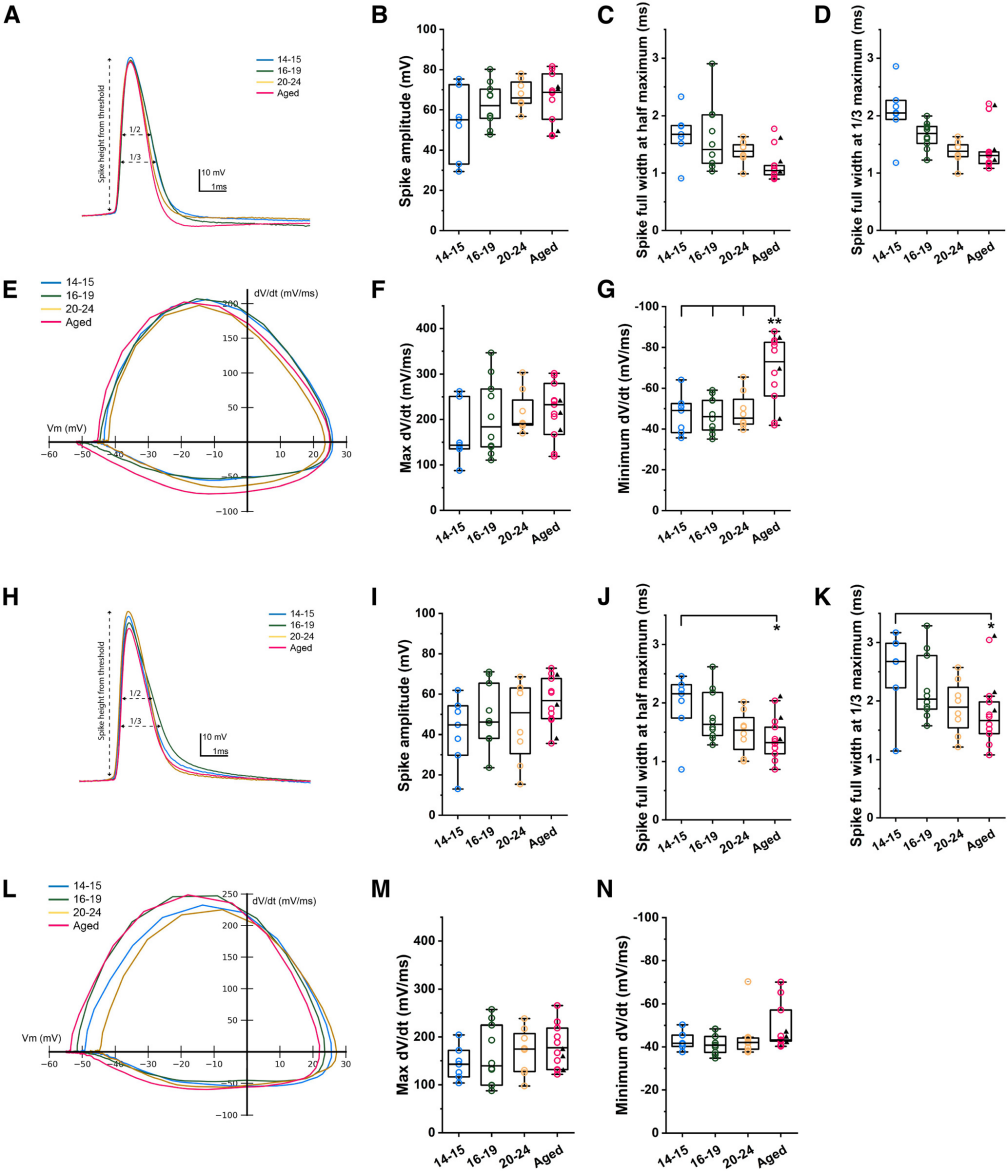
Spike properties of CA1 pyramidal cells in young and aged rats in ACSF and with carbachol. ***A–G***, Spike properties measured without carbachol. ***A***, Example voltage traces of action potential from all four groups. ***B***, Spike amplitude. ***C***, Spike half width. ***D***, Spike width at one-third of spike amplitude. ***E***, Examples of membrane potential slope versus membrane potential from all four groups of cells. ***F***, Maximum slope of spike. ***G***, Minimum slope of spike (Tukey’s *post hoc* test, P14–P15 vs Aged, *p* = 0.0062; P16–P19 vs Aged, *p* = 0.0021; P20–P24 vs Aged, *p* = 0.0083). ***H–N***, Spike properties measured with carbachol (10 μm). ***H***, Example voltage traces of action potential from all four groups. ***I***, Spike amplitude. ***J***, Spike half width (Tukey’s *post hoc* test, P14–P15 vs Aged, *p* = 0.027). ***K***, Spike width at one-third of spike height (Tukey’s *post hoc* test, P14–P15 vs Aged, *p* = 0.0349). ***L***, Examples of membrane potential slope versus membrane potential from all four groups of cells. ***M***, Maximum slope of spike. ***N***, Minimum slope of spike.

Since aging has been shown to affect spike properties, and persistent firing is believed to depend on the calcium influx during spikes, we compared spike properties among the four groups both with and without carbachol. As shown in Figure 3*A*,*B*, spike amplitude was not significantly different in any of the groups (one-way ANOVA, *p* = 0.0984, *F*_(3,31)_ = 2.28; Fig. 3*B*). Spike widths measured at the half and a third spike height were both narrower in the aged group showing a decreasing trend with development (Welch’s one-way ANOVA, *p* = 0.0422, *F*_(3,15.78)_ = 3.45, one-way ANOVA, *p* = 0.0412, *F*_(3,31)_ = 3.09, respectively; Fig. 3*C*,*D*). In the slope domain, the rising slope was similar but the falling slope was steeper in the aged group compared with all the young groups (one-way ANOVA, *p* = 0.278, *F*_(3,31)_ = 1.34, one-way ANOVA, *p* = 0.0009, *F*_(3,31)_ = 7.16, respectively; Fig. 3*F*,*G*). These results are in line with the early developmental increase of voltage-gated potassium channels during the first month of life that is associated with the refinement of the intrinsic membrane properties, yielding a progressive decrease in the spike width (Spigelman et al., 1992; Dougherty, 2020).

In the presence of carbachol, spike widths at the half and a third spike height were narrower in the aged group (one-way ANOVA, *p* = 0.0237, *F*_(3,31)_ = 3.62, one-way ANOVA, *p* = 0.0339, *F*_(3,31)_ = 3.28, respectively; Fig. 3*J*,*K*) similarly to the condition without carbachol.

### Persistent firing

**Figure 4.**
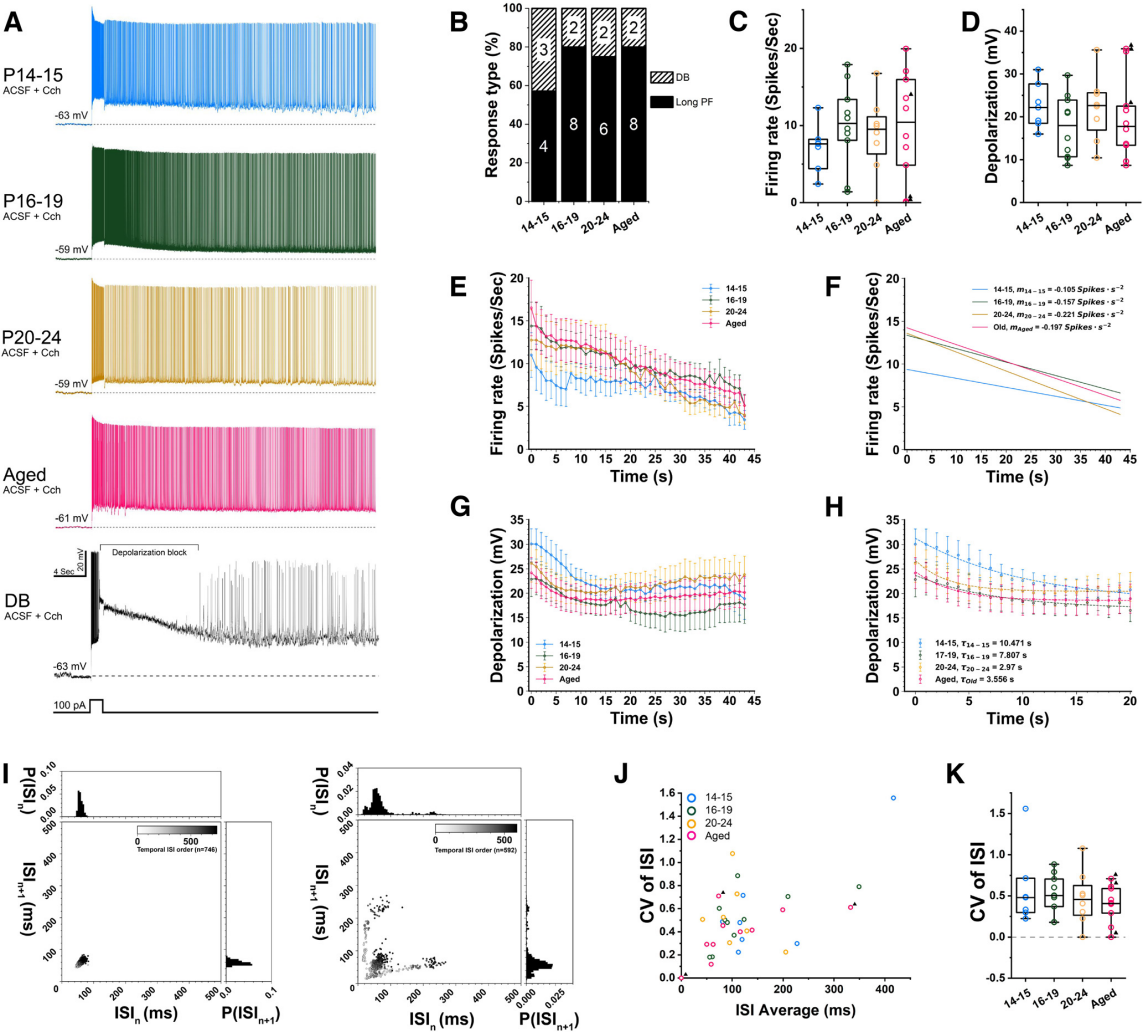
Comparison of persistent firing among different age groups. ***A***, Examples of persistent firing observed in all age groups in the presence of carbachol (10 μm). The fifth voltage trace from the top indicated with DB is an example of depolarization block response also in presence of carbachol (10 μm). The depolarization block state is marked with a bracket. The bottom trace shows injected current (2 s, 100 pA). ***B***, Percentages of cells that showed persistent firing. All cells responded with one of the three types of persistent firing. Long PF: persistent firing lasting >30 s. Short PF: persistent firing self-terminating within 30 s. DB: persistent firing which included depolarization block. ***C***, Frequency of persistent firing measured as the average over the 45 s after the current stimulation offset. ***D***, Membrane depolarization during persistent firing measured as the difference from the baseline potential. ***E***, Average time course of frequency of persistent firing. ***F***, Linear fits of persistent firing frequency in ***E***. *m* represents the slope for each age groups. ***G***, Average time course of membrane depolarization during persistent firing. ***H***, Exponential fits of depolarization shown in G. *τ* represents the decay time constant for each age groups. ***I***, Return map of interspike interval (ISI) taken from two example cells that displayed a regular firing (left) and bursting firing (right) pattern during persistent firing. ***J***, Coefficient of variation (CV) of ISI as the function of average ISI that indicates the degree of burst spiking from all cells. ***K***, Comparison of CV of ISI among four groups of cells.

Next, we tested whether persistent firing in young and aged rats was different. Measurements were done in the same sets of cells as in the previous figures. Persistent firing was tested using a brief current injection stimulation (2 s, 100 pA; Fig. 4*A*) in the presence of the cholinergic receptor agonist carbachol (10 μm). The membrane potential was adjusted to slightly below the level where spontaneous spikes occur, before the brief current stimulation, similarly to previous reports of this type of persistent firing (Egorov et al., 2002; Knauer and Yoshida, 2019). This enabled us to compare the ability of individual cells to engage in persistent firing independently from the native membrane potentials that differ from cell to cell. This membrane potential before the current stimulation is referred to as baseline membrane potential. As reported previously (Knauer et al., 2013), persistent firing was not observed when carbachol was not present.

As shown in Figure 4*A*, persistent firing was observed in all four groups of cells. When the 2-s current stimulus was terminated, membrane potential stayed depolarized compared with the baseline potential and repetitive action potentials were observed. In all groups, ∼80% of neurons tested responded with long-lasting persistent firing that lasted for longer than 30 s (Fig. 4*B*, black). In a minority of cells (∼20%), persistent firing included a period of time when the membrane potential was above the spike threshold but action potential firing was absent. This type of persistent activity was referred to as depolarization block response (DB; Fig. 4*B*, striped). These measurements indicate that both the observed types of persistent firing and how often they occur are similar in all four age groups.

Next, we compared the frequency of persistent firing and membrane depolarization during persistent firing which were measured as the average of the 0- to 45-s period after the termination of the stimulus (Fig. 4*C*,*D*). In this analysis, the frequency of persistent firing was simply an average frequency of firing during these 45 s. The depolarization was measured as the difference between the baseline membrane potential and the average membrane potential during the 45 s period. We found that both the firing frequency (one-way ANOVA, *p* = 0.6949, *F*_(3,31)_ = 0.48) and depolarization (one-way ANOVA, *p* = 0.5552, *F*_(3,31)_ = 0.71) were not significantly different between the four groups, while there was a tendency that the youngest group showed a somewhat lower persistent firing frequency. These results suggest that persistent firing in all four groups is quantitatively similar when averaged values are compared.

Next, we asked whether the time course of persistent firing was different among the four groups by plotting the frequency and depolarization of persistent firing on the time axis (Fig. 4*E–H*). We found that the frequency of persistent firing goes down linearly for the duration of the recording (Fig. 4*E*,*F*). When the rate of decrease in firing frequency was compared among the four groups, there were no significant differences (repeated measure two-way ANOVA, Geisser–Greenhouse correction, *p* = 0.9872, *F*_(129,1333)_ = 0.73). However, there was a tendency that the youngest group (P14–P15) had a lower frequency initially causing a slower decrease in firing rate over time. On the other hand, membrane depolarization during persistent firing went down more nonlinearly where most of the drop occurred within the initial 10 s (Fig. 4*G*,*H*). Hence, the firing frequency continued to decrease while the depolarization remained stable after this initial drop of membrane potential. We noticed that the youngest group started off the persistent firing with a more depolarized potential than the other groups (Fig. 4*G*). When the initial decrease of membrane depolarization was fitted with an exponential function, the time constant of the decay was the longest in the two youngest groups (Fig. 4*H*). However, the values in the aged group were very similar to the P20–P24 group, and there was no statistically significant difference between the young and aged groups (Tukey’s post hoc test, *p* ≥ 0.3197). These analyses, therefore, suggest that CA1 pyramidal cells in very old rats can maintain the ability to respond with robust persistent firing similar to those in young groups.

Cells from rats that did not go through the behavioral training showed the highest depolarization values (Fig. 4*D*). In fact, two of these cells responded with a depolarization block during which the membrane potential was so depolarized that action potentials could not be elicited (Fig. 4*A*, bottom). As we and others have shown in the past, such depolarization block response is a strong form of persistent activity often observed when relatively strong cholinergic activation is used (C. Tai et al., 2011; Knauer and Yoshida, 2019). Since the number of cells that were sampled from these animals was small, we do not draw any conclusion here. However, possible effects of behavioral training are discussed further in Discussion.

We also noticed that some neurons were firing in bursts during persistent firing while others were regularly spiking. Figure 4*I* shows two example return maps of interspike intervals (ISIs) from cells that responded with regular spiking (left) and bursts (right) during persistent firing. To test whether there was an age-dependent difference in burst firing, we calculated the covariance (CV) of ISI (one-way ANOVA, *p* = 0.6048, *F*_(3,31)_ = 0.62; Fig. 4*J*,*K*). As seen in these figures, we did not find a significant difference in burst firing likelihood among the four groups of cells we compared.

To test whether persistent firing was induced in similar conditions in all groups, we also compared the baseline membrane potential. Baseline membrane potential was not significantly different (P14–P15: −58.36 ± 1.42 mV; P16–P19: −59.88 ± 1.19 mV; P20–P24: −58.19 ± 1.35 mV; Aged: −61.68 ± 0.90 mV; one-way ANOVA, *p* = 0.158, *F*_(3,31)_ = 1.85). In addition, firing rate during the 2 s stimulation was not significantly different (P14–P15: 28.64 ± 1.94 spikes/s; P16–P19: 27.35 ± 2.79 spikes/s; P20–P24: 27.93 ± 3.92 spikes/s; Aged: 23.40 ± 3.82 spikes/s; Kruskal–Wallis, *p* = 0.7388), indicating that cells responded similarly during the stimulation.

In addition, we compared the anatomic location of neurons used in these analyses. We previously reported that the DB likelihood increases toward the proximal side within CA1 (Knauer and Yoshida, 2019). Since the aged group neurons were entirely sampled from the distal half, only cells recorded from the distal half of CA1 were used for the young groups as well. Therefore, the average proximodistal locations of neurons from all four groups were similar (P14–P15: 79 ± 4%; P16–P19: 79 ± 4%; P20–P24: 71 ± 6%; Aged: 71 ± 4%; one-way ANOVA, *p* = 0.3614, *F*_(3,31)_ = 1.11). Also along the dorsoventral axis, which was not controlled, cell locations did not differ between the age groups (P14–P15: 2.9 ± 0.4 mm; P16–P19: 3.1 ± 0.4 mm; P20–P24: 3.2 ± 0.4 mm; Aged: 3.7 ± 0.4 mm; one-way ANOVA, *p* = 0.5434, *F*_(3,31)_ = 0.73).

### Spike afterhyperpolarization potential and persistent firing

We next examined the relationship between the medium and slow spike afterhyperpolarization potentials (mAHP and sAHP) and persistent firing. mAHP and sAHP have been reported to affect persistent firing and are shown to be modulated by aging and behavioral tasks (Disterhoft et al., 1986; Matthews et al., 2009; Oh et al., 2010). The AHPs were measured using two different protocols.

**Figure 5.**
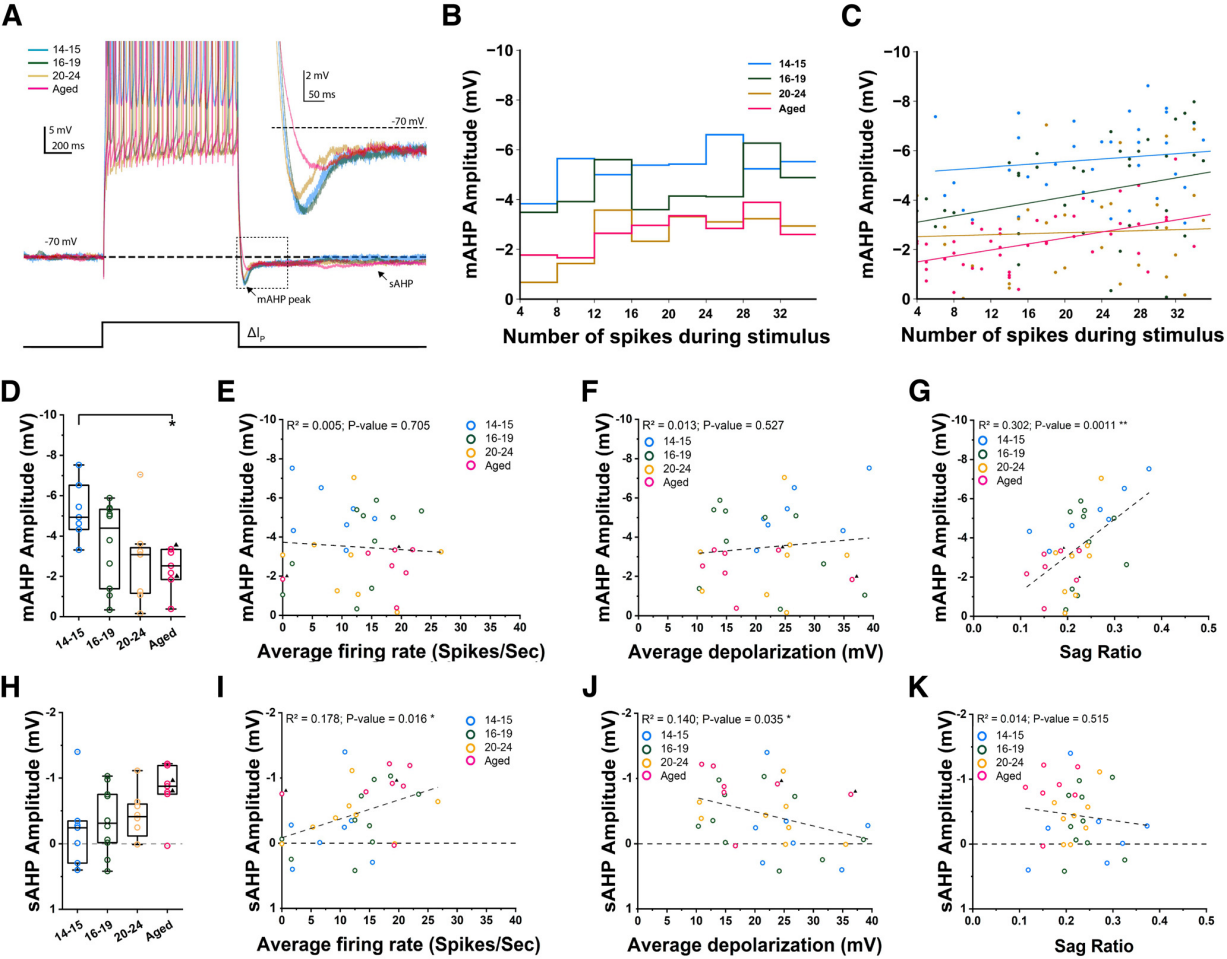
Spike afterhyperpolarization potentials (AHP) at −70 mV and persistent firing. ***A***, Example voltage traces from which mAHP and sAHP were measured. Inset, shows a magnified view of the area indicated by the dashed square. Bottom trace shows injected current (1 s, variable amplitude). ***B***, Comparison of mAHP amplitudes for all four groups of cells (Tukey’s *post hoc* test, P14–P15 vs Aged, *p* = 0.0278). ***C***, mAHP measured with different current injection levels that elicited different numbers of spikes. ***D***, Same as ***C*** but for individual neurons. Lines are linear fittings showing the correlation between mAHP and number of spikes during stimulus. ***E***, Correlation between mAHP amplitude and frequency of persistent firing. ***F***, Correlation between mAHP amplitude and membrane depolarization during persistent firing. ***G***, Correlation between mAHP amplitude and sag ratio measured from a baseline of −70 mV. ***H***, Comparison of sAHP amplitudes for all four groups of cells. ***I***, Correlation between sAHP amplitude and frequency of persistent firing. ***J***, Correlation between sAHP amplitude and membrane depolarization during persistent firing. ***K***, Correlation between sAHP amplitude and sag ratio measured from a baseline of −70 mV.

First, mAHP and sAHP were measured after a brief current injection (1 s) from a fixed membrane potential of −70 mV (Fig. 5*A*). The amplitude of the current was increased in steps from 50 to 400 pA and both mAHP and sAHP were measured multiple times in each cell. The mAHP amplitudes measured from the first current step that induced >15 spikes decreased with age and the difference between the youngest group and the old group was significant (one-way ANOVA, *p* = 0.0052, *F*_(3,28)_ = 5.16; Fig. 5*D*). The numbers of spikes induced by this current step were not different among the four groups (one-way ANOVA, *p* = 0.6954, *F*_(3,28)_ = 0.48). This decreased mAHP amplitude with age was a general trend across a wide range of induced spikes as shown in Figure 5*B*,*C* (linear regression; P14–P15: *p* = 0.048, *F*_(1,82)_ = 4.00; P16–P19: *p* = 5.7e-4, *F*_(1,91)_ = 12.75; P20–P24: *p* = 0.8452, *F*_(1,42)_ = 0.038; Aged: *p* = 2.3e-4, *F*_(1,50)_ = 15.77). We then asked whether the mAHP measurements correlated with persistent firing using the mAHP values shown in Figure 5*D*. We did not observe a clear correlation between the mAHP amplitude and the strength of persistent firing (linear regression; *p* = 0.705; Fig. 5*E*) or depolarization (linear regression; *p* = 0.527; Fig. 5*F*). However, mAHP showed a significant correlation with the sag ratio (linear regression; *p* = 8.94e-5; Fig. 5*G*), suggesting a substantial contribution of the h-current to the mAHP at membrane potentials close to −70 mV (Gu et al., 2005). AHP measurement in the carbachol condition was not analyzed because AHP amplitude was often near zero or positive because of the development of an after-depolarization potential. We then measured sAHP using the membrane potential 1 s after the offset of the current injection in the same set of recordings. As shown in Figure 5*H*, we did not observe significant differences among the four groups (one-way ANOVA, *p* = 0.1336, *F*_(3,28)_ = 2.02; Fig. 5*H*). However, we did observe correlations between the sAHP amplitude and persistent firing (linear regression; *p* = 0.016; Fig. 5*I*) and depolarization (linear regression; *p* = 0.035; Fig. 5*J*).

**Figure 6.**
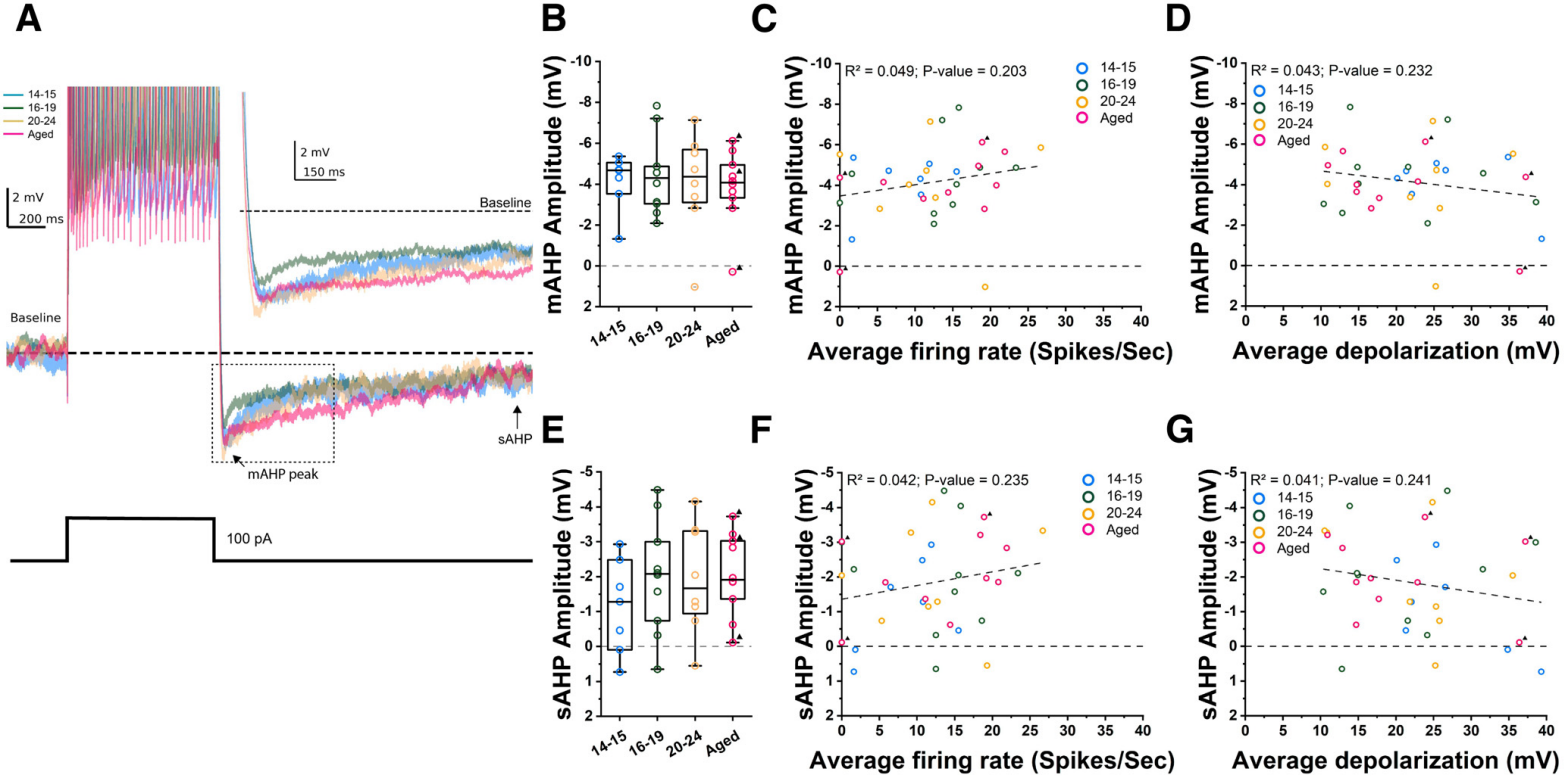
Spike afterhyperpolarization potentials (AHP) at baseline potential and persistent firing. ***A***, Example voltage traces from which mAHP and sAHP were measured. Inset, Magnified view of the area indicated by the dashed square. Bottom trace shows injected current (2 s, 100 pA). ***B***, Comparison of mAHP amplitudes for all four groups of cells. ***C***, Correlation between mAHP amplitude and frequency of persistent firing. ***D***, Correlation between mAHP amplitude and membrane depolarization during persistent firing. ***E***, Comparison of sAHP amplitudes for all four groups of cells. ***F***, Correlation between sAHP amplitude and frequency of persistent firing. ***G***, Correlation between sAHP amplitude and membrane depolarization during persistent firing.

Second, to evaluate AHPs in conditions closer to those where persistent firing was tested, we measured AHPs using the same protocol we used for the induction of persistent firing. In these recordings, a brief current injection (100 pA for 2 s) was applied at the membrane potential just below the spiking threshold. With this measurement, the mAHP amplitudes between the young and old groups (one-way ANOVA, *p* = 0.9357, *F*_(3,31)_ = 0.14; Fig. 6*A*,*B*) were not different among any of the four groups. In addition, it shows that the three AHP measures from the two untrained rats were scattered and did not appear to differ from those measured from the trained rats. In addition, we did not find a clear correlation between the mAHP amplitudes and the strength of persistent firing (linear regression; *p* = 0.203; Fig. 6*C*) or depolarization (linear regression; *p* = 0.232; Fig. 6*D*). sAHP measured from these recordings were also similar among the four groups (Fig. 6*E*) and we did not find a clear correlation with the strength of persistent firing (linear regression; *p* = 0.235; Fig. 6*F*) or depolarization (linear regression; *p* = 0.241; Fig. 6*G*). The lack of mAHP amplitude difference and the lack of correlation between sAHP and persistent firing with this protocol might be because of the extended period of depolarization and more depolarized potential. In summary, we did not observe an age-dependent increase of mAHP or sAHP amplitude in our data, contrary to some previous reports (Matthews et al., 2009; Lin et al., 2020). Nevertheless, our data suggests that sAHP may modulate persistent firing in rat CA1 pyramidal cells.

### Depolarizing current and membrane capacitance correlate in carbachol

**Figure 7.**
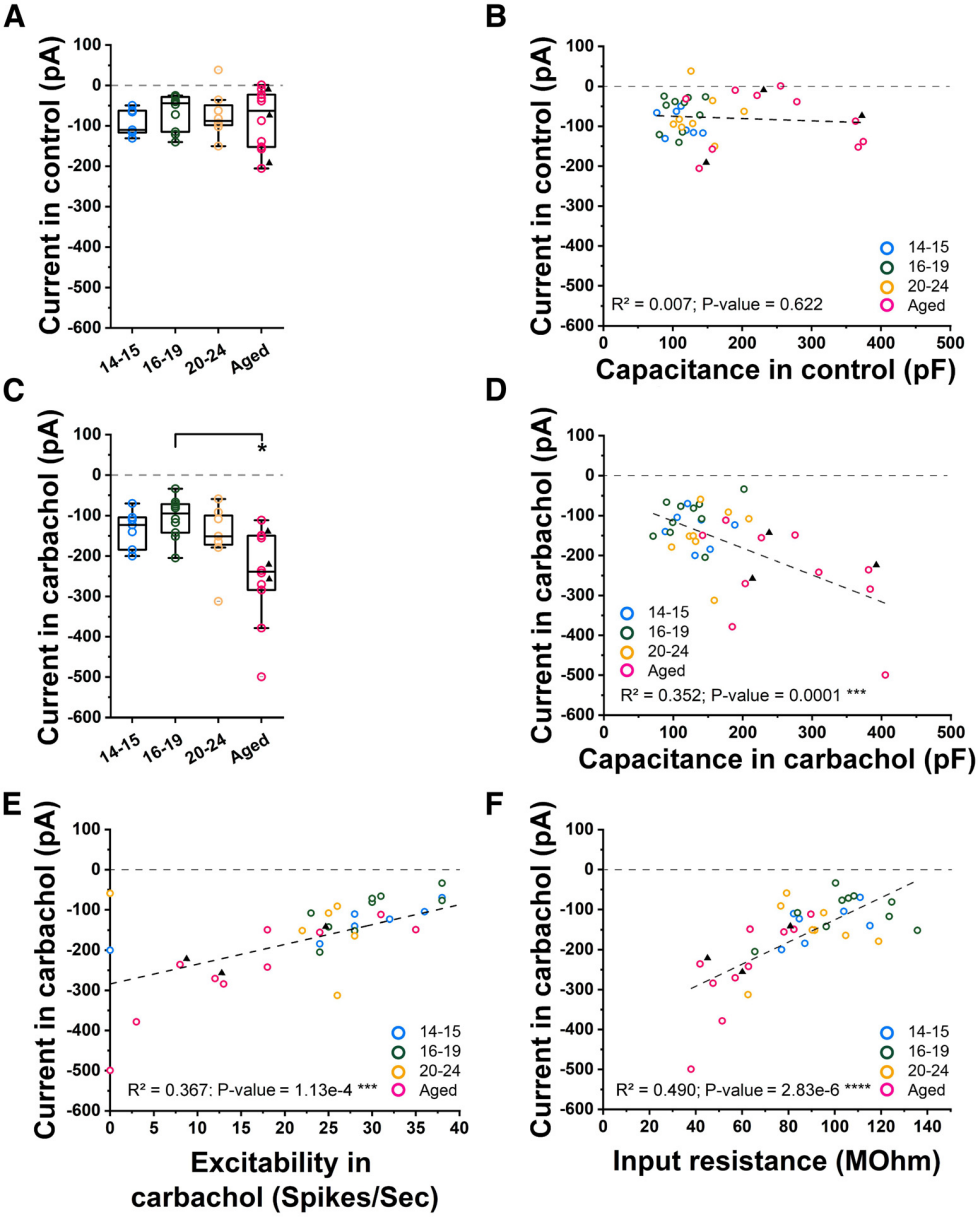
Cholinergically induced depolarization current. ***A***, Comparison of the injected current to maintain cells at −70 mV in control (ACSF) for all four groups of cells. ***B***, Correlation between the injected current to maintain cells at −70 mV in control (ACSF) and capacitance in control. ***C***, Comparison of the injected current to maintain cells at −70 mV in carbachol for all four groups of cells. ***D***, Correlation between the injected current to maintain cells at −70 mV in carbachol and capacitance in carbachol. ***E***, Correlation between the injected current to maintain cells at −70 mV in carbachol and excitability in carbachol. ***F***, Correlation between the injected current to maintain cells at −70 mV in carbachol and input resistance in carbachol.

It has been shown that application of carbachol depolarizes the membrane potential through the changes in at least two types of ionic current: suppression of potassium current such as the M-current, and activation of cationic current such as the CAN current (Benardo and Prince, 1982; Gähwiler and Dreifuss, 1982; Halliwell and Adams, 1982; Cole and Nicoll, 1983). As we and others have reported (for review, see Yoshida et al., 2012), cholinergically induced persistent firing is supported by a CAN current which is a membrane cationic current that depolarizes the membrane potential. Since the aged group neurons had lower intrinsic excitability, it would require a larger CAN current for them to respond with persistent firing with similar strength to the young groups. To address this possibility, we next measured the current injection required to hold the membrane potential at −70 mV both in control condition without carbachol (one-way ANOVA, *p* = 0.7442, *F*_(3,31)_ = 0.41; Fig. 7*A*) and in carbachol (Welch’s one-way ANOVA, *p* = 0.0279, *F*_(3,16.37)_ = 3.92; Fig. 7*C*). Since the CAN current is partially activated in carbachol even at the resting membrane potential (Haj-Dahmane and Andrade, 1996; Zhang et al., 2011), this measure should reflect the amount of activated CAN current. The potassium current which is suppressed by carbachol will also partially contribute to this current. However, its contribution should be smaller at −70 mV where the M-current is in theory inactive (Hönigsperger et al., 2015). We found that this current is larger in the aged group only in the presence of carbachol compared with the two youngest groups (Tamhane’s T2, P14–P15 vs Aged *p* = 0.0992, P16–P19 vs Aged *p* = 0.0274; Fig. 7*C*), indicating a possibility that CAN current is larger in the aged group (Fig. 7*C*).

We have shown that the membrane capacitance, which reflects the membrane area, is larger in the aged cells in the previous section (Figs. 1*I*, 2*I*). To examine whether the amplitude of this current is in proportion to the membrane area, we tested the correlation between the current and the membrane capacitance (Fig. 7*B*,*D*). We found a significant correlation only in carbachol indicating that this carbachol-activated current is proportional to the larger area observed in aged cells (linear regression; *p* = 0.0001, *F*_(1,33)_ = 17.93; Fig. 7*D*). This suggests that the density of current per unit membrane area is similar to the young groups. Furthermore, we found that this current correlates with the intrinsic excitability (linear regression; *p* = 0.0001, *F*_(1,33)_ = 19.16; Fig. 7*E*) and input resistance (linear regression; *p* = 2.83e-6, *F*_(1,33)_ = 31.76; Fig. 7*F*) measured in carbachol in individual cells. This supports the idea that the amount of CAN current could compensate for the lower excitability and lower input resistance of aged neurons to achieve a similar amount of depolarization to support persistent firing.

## Discussion

Motivated by the potential role of intrinsic persistent firing as a cellular mechanism underlying working memory-related persistent firing and cholinergically mediated age-dependent decline of cognitive performance, in this paper, we compared persistent firing in young rats (P14–P24) with old rats (24–27 months) in an *in vitro* preparation. Surprisingly, persistent firing in the old group we tested was as strong as in the young groups, while the analysis of basic membrane properties indicated that aging significantly affected properties such as the intrinsic excitability, input resistance, sag ratio, and membrane capacitance of aged neurons. In contrast to some of the previous reports, we did not observe increased AHPs in the aged group, while the sAHP amplitude was weakly correlated with the strength of persistent firing. Finally, we demonstrated that comparable persistent firing in the aged group neurons could be supported by the increased CAN current that counteracted the low excitability of these cells.

### Persistent firing in aged animals

Persistent firing observed *in vivo* has been proposed to be a cellular correlate of working memory and temporal bridging both in humans and animals (Major and Tank, 2004; Reboreda et al., 2018; Lin et al., 2020). While the prevailing hypothesis is that persistent firing could be supported by excitatory synaptic connections among neurons, we and others have been providing evidence suggesting that individual neurons could support persistent firing (Klink and Alonso, 1997; Egorov et al., 2002; Knauer et al., 2013). These studies were mainly performed *in vitro* using young or adult animals, and they demonstrate the involvement of ionic mechanisms such as the TRPC, h-current, and hERG channels (Zhang et al., 2011; Thuault et al., 2013; Cui and Strowbridge, 2018; Arboit et al., 2020). Thus far, data on the development of persistent firing and the effect of aging on it still remain scarce.

Existing literature suggests that persistent firing emerges with the development of the cholinergic system in the entorhinal cortex in juvenile rats (Reboreda et al., 2007), and aging impairs persistent firing in the lateral entorhinal cortex (Lin et al., 2020). In the hippocampus, while persistent firing has been reported in CA1 (Knauer et al., 2013), CA3 (Jochems and Yoshida, 2013), and the dentate gyrus (Larimer and Strowbridge, 2010), no study has so far focused on development or aging. Our results indicate that persistent firing in the aged group is as strong as that in the young rats (Fig. 4). Not only were the average firing frequency and depolarization similar among the four groups, but the ratio of neurons that responded with persistent firing, and the temporal course of persistent firing (Fig. 4*E–H*) was also similar.

The fact that persistent firing did not differ among the three young groups is generally in agreement with the previous observation from entorhinal neurons in developing rats (Reboreda et al., 2007). They reported that persistent firing was not significantly different in their 16- to 19- and 21-to 23-d-old group rats, which were within the range of age we tested. However, it was somewhat surprising for us to find that persistent firing in the aged group was also similar to the young groups for the following reasons. First, *in vivo* electrophysiological recordings from aged monkeys have shown decreased persistent firing during a working memory task (Wang et al., 2011). Second, lower intrinsic excitability of CA1 neurons is reported in aged rabbits (Moyer et al., 2000), and the same group has recently demonstrated that persistent firing is reduced in the lateral entorhinal cortex of aged rats (Lin et al., 2020). Third, the cholinergic stimulation which is required for the induction of persistent firing has been shown to degrade in aging (Ballinger et al., 2016). In the following sections, we will discuss these in more detail.

### Intrinsic excitability and persistent firing

While intrinsic excitability is one of the fundamental parameters that control the firing behavior of individual neurons, a clear link between intrinsic excitability and persistent firing has not been demonstrated. In our analysis, the intrinsic excitability was the lowest in the aged rats (Fig. 1*B*). This is in line with the literature from rabbit CA1 neurons which reported that aged CA1 neurons fire fewer action potentials in response to a brief current injection than young neurons (Moyer et al., 1992, 2000). However, to maintain cellular homeostasis, the intrinsic excitability is also tuned by multiple factors such as the input resistance, spike threshold, and the magnitudes of AHPs. As for the input resistance (Fig. 1*C*), the lower value we found in the aged group compared with the young groups is in line with higher input resistances found in young groups in CA1 pyramidal cells from rats (Cherubini et al., 1989; Spigelman et al., 1992) and rabbits (Schwartzkroin and Kunkel, 1982; Moyer et al., 1992), and with the maintained input resistance between adult and aged animals (Pitler and Landfield, 1990; Matthews et al., 2009). Similarly, these same studies indicate that input resistance and membrane time constant are stable around days P15–P30, which is in line with the fact that we did not find many significant differences among the three young groups.

Changes in sag ratio may also reflect mechanisms which influence cellular excitability (Magee, 1998; Fan et al., 2005). The hyperpolarization-activated current (I_h_) that underlies the sag potential develops in the first three postnatal weeks, and it is comparable to adults by P20 days (Vasilyev and Barish, 2002). The decrease of the sag ratio we observed over age is in line with decreased I_h_ in aged gerbils which reflected a reduction in HCN1 and 2 in the hippocampus (Lee et al., 2019). The smaller sag ratio observed in the aged group both with and without carbachol (Figs. 1*G*, 2*G*) in our study should in theory have counteracted the decreased input resistance of this group (Figs. 1*C*, 2*C*) tempering the decrease in intrinsic excitability (Figs. 1*B*, 2*B*).

### Spike-afterhyperpolarization potentials (AHP) and persistent firing

Aging is commonly associated with a decrease in neuronal excitability of CA1 neurons (Oh et al., 2016). Previous research indicated that this age-related decrease of excitability is associated with an increase of the postburst slow and medium components of the Ca^2+^-dependent K^+^-mediated afterhyperpolarization (AHP). The magnitude of AHP in CA1 pyramidal cells both in rats and rabbits is shown to increase with aging (Power et al., 2002; Matthews et al., 2009).

Interestingly, while we did find reduced excitability of aged neurons in CA1 (Fig. 1*B*), we did not see any significant increase in mAHP or sAHP amplitude with age (Figs. 5, 6). In fact, we observed a decrease in mAHP amplitude when we used a current injection of 1 s which elicited a controlled number of spikes (Fig. 5*B–D*). We employed a 2-s-long stimulus of 100 pA to evaluate AHPs in multiple ways (Fig. 6). With this method, the mAHP and sAHP were not significantly different in all age ranges although the number of spikes during the current step was significantly lower in aged rats compared with P16–P19 and P20–P24 cells (one-way ANOVA, *p* = 0.0089; data not shown). It was also noticed that the amplitudes of mAHP were larger in this 2-s-long protocol in most of the groups except for the youngest compared with those in the 1-s-long protocol. This could be because of more accumulation of calcium during the longer period of depolarization and more activation of calcium-dependent K^+^ channels. This also indicates that the reason why we did not see a difference in mAHP amplitude with the 2-s-long method could be because of the saturation of mAHP.

The mAHP and sAHP amplitude contribute to intrinsic excitability in CA1 neurons by suppressing repetitive firing (H.M. Wu et al., 2003; for review, see Oh et al., 2016). In fact, our analysis indicated that spike frequency adaptation was larger in the youngest group (Fig. 1*E*) which showed the largest mAHP amplitude (Fig. 5*D*). However, while we observed decreased intrinsic excitability of aged neurons, we did not observe increased AHP amplitudes in this group. This indicates that the lower intrinsic excitability of the aged group cells was because of other properties such as the lower input resistance in this group (Fig. 1*C*).

As for why we did not observe age-dependent increases in AHP, which are reported by some studies, the strain of rats used in each study should be considered. It has been reported that age-dependent changes in AHP vary from strain to strain (Potier et al., 1993). Specifically, that AHP amplitude nonsignificantly decreased in Wistar rats, showed no change in Fisher 344 rats (Potier et al., 1993), and increased in both Sprague Dawley rats (Potier et al., 1992) and in F1 hybrid Fischer 344 × Brown Norway rats (Matthews et al., 2009) with aging. In aged Long–Evans rats, animals with normal learning ability showed mAHP and sAHP similar to young animals, while learning impaired animals showed significant decreases in both (Severin et al., 2020). Our results, which indicate no strong modulation of AHP amplitudes by aging alone in Long–Evans rats, add a new data point to the previous reports.

Another reason could be the involvement of the rats we used in this study in prior behavioral training. Successful learning of certain tasks has been shown to reduce the AHP amplitude and spiking accommodation, resulting in an increase in neuronal excitability in CA1 (Matthews et al., 2009). Among the aged group neurons we used, seven out of 10 cells were obtained from rats that went through behavioral training (water maze, see Materials and Methods). Therefore, we cannot exclude the possibility that this behavioral training reduced the AHPs in aged cells. However, comparing three neurons that came from untrained animals with other cells speaks against this view. Cells from untrained animals displayed on average a similar level of neuronal excitability as cells recorded from trained animals (Fig. 1). The AHP amplitudes from untrained animals were on average not significantly different from the AHP from trained animals (Figs. 5, 6).

It has been described that many factors other than behavioral training might influence AHP properties of hippocampal neurons, such as housing conditions (Kumar and Foster, 2007). Since our aged rats (as well as the young rats) were group housed, and were manipulated by experimenters, these factors might have contributed to maintaining AHP amplitudes similar to the young groups.

Additionally, Oh et al. (2003) have shown that AHP is decreased in dorsal CA1 after learning. Since we recorded from both dorsal and ventral hippocampus, such effects were plausibly diluted in our study. Plus, sampling differences along the proximodistal axis in our data and publications from other groups could be a source of different results.

Finally, the lack of modulation of persistent firing by aging in our study compared with previously mentioned data from the lateral entorhinal cortex (Lin et al., 2020) could have stemmed from this lack of AHP modulation in our dataset. While Lin and colleagues (2020) have shown that mAHP was larger in the aged nonlearner group, we observed neither the mAHP reduction nor the correlation between mAHP amplitude and strength of persistent firing (Figs. 5, 6). We however, observed that sAHP was weakly correlated with persistent firing when sAHP was measured at −70 mV (Fig. 5). This may indicate that sAHP but not mAHP modulates persistent firing in CA1 pyramidal cells. However, lack of this correlation at more depolarized membrane potential (Fig. 6) closer to the condition where persistent firing was tested, and the lack of sAHP modulation by aging, are both in line with the similar strength of persistent firing we observed in all groups of cells. It is interesting to point out here that persistent firing in CA1 neurons seems to be different from that observed in the lateral entorhinal cortex. For instance, persistent firing in CA1 cells started off strong and slowly decayed over time (Fig. 4*E*), while persistent firing started weakly and increased in frequency over time in the lateral entorhinal cortex. Since the initial few seconds of persistent firing is when AHP induced by the current step is strong, this could indicate a weaker effect of AHP on persistent firing in CA1 neurons. Therefore, the effect of AHP on persistent firing might depend on the cell types.

Additionally, as shown for the data set out of which the young group was drawn (Knauer, 2016), there was no significant difference between males and females, either in intrinsic membrane properties or persistent firing properties. Therefore, it is unlikely that the strength of persistent firing in the aged group would be different from younger groups if we limited our young groups to male rats only. It should, however, be pointed out that we cannot exclude the possibility that the relatively high concentration of carbachol we used in our study could mask a hypothetical difference in persistent firing between the young and aged groups at a lower level of cholinergic activation.

### Potential involvement of TRP channels

Despite changes in intrinsic membrane properties during rat development, CA1 cells tightly maintained the proportion of neurons capable of persistent firing as well as the robustness of the persistent firing frequencies and depolarization plateau from P14 to advanced ages (Fig. 4). A substantial body of evidence points toward a role of the Ca^2+^-activated nonselective cation channels (CAN) permeable to monovalent cations in supporting persistent firing in the hippocampus (Knauer et al., 2013; Reboreda et al., 2018). While the CAN current might be mediated by different members of the TRP family following cholinergic activation, recent studies highlighted the role of TRPC4 and TRPC5 channels in supporting the CAN current in CA1 (C. Tai et al., 2011; Arboit et al., 2020). Information in regard to hippocampal TRPC channel expression during development is still scarce, yet *in situ* hybridization experiments revealed that a peak in the expression of TRPC4 and TRPC5 channels is detected at P14 (Y. Tai et al., 2008). Such expression of TRPC4 and TRPC5 channels at P14 seems to be consistent with the frequency of persistent firing and depolarization plateau amplitudes that reached adult-like levels in the P14–P15 group (Fig. 4).

The fact that the aged group cells responded with persistent firing as strong as the young group despite the lower intrinsic excitability might suggest that the old group had a stronger CAN current. To give an insight into this possibility, we measured carbachol-activated current and found that this current is larger in the aged group (Fig. 7*C*). The significant positive correlation of this current with the membrane capacitance (Fig. 7*D*) indicates that the density of the current in a unit membrane area could be similar in cells from different age groups. And, the negative correlation between this current and the excitability in individual cells (Fig. 7*E*) suggests that the CAN current could excite individual cells to a certain level despite their different intrinsic excitabilities. While the induction of persistent firing was triggered by an injection of current from the pipette, the membrane CAN current is the main drive once the injection of the current is terminated and persistent firing is initiated. Our data suggests that aged cells could support similar persistent firing by having sufficiently larger CAN current than young cells to compensate for their lower intrinsic excitability.

### Cholinergic degradation in aging

Cholinergic projections from the medial septal area to the hippocampus have been shown to have prominent roles in working memory, learning, synaptic plasticity and attention (Hasselmo, 2006). Moderate degeneration of cholinergic functions has been generally attributed to age-related decline of memory and to a further extent, to neurologic disorders such as Alzheimer’s disease or dementia (Schliebs and Arendt, 2011). In line with this, it has been reported that persistent firing *in vivo* is reduced in aged monkeys during a working memory task (Wang et al., 2011). However, it remains unclear how cholinergic degradation affects persistent firing *in vivo*.

Effects of cholinergic degradation at the receptor levels have been studied intensively. Cholinergic transmission, as well as postsynaptic membrane response to 0.3 μm carbachol are reduced in 18- to 20-month-old aged rats compared with young one- to two-month-old rats (Taylor and Griffith, 1993). These changes were not accompanied by a global decline in muscarinic receptor functions (Taylor and Griffith, 1993), but rather by a decline of the level of acetylcholine in the hippocampus in aged, relative to young adult, rats (Scali et al., 1995). In agreement with these results, Reboreda et al. (2007) demonstrated that the mRNA levels of the M1 subtype of muscarinic receptors in the entorhinal cortex, which is crucial for persistent firing, stayed constant after postnatal day 12. In addition, there is evidence that acetylcholine release is reduced in aged animals (C.F. Wu et al., 1988; Chang and Gold, 2008). On the other hand, there is evidence for compensatory mechanisms for this reduction of acetylcholine release. For example, M2 receptor density is higher in the aged group than in the young group in (Aubert et al., 1995). Together, these suggest that while acetylcholine release could be impaired in the aged brain, some downstream mechanisms which might support persistent firing could be kept relatively intact. Our results, which indicate intact persistent firing in CA1 pyramidal cells, suggest one mechanism of this sort.

This view is in line with the fact that cholinergic enhancement using acetylcholinesterase inhibitors rescues the memory performance in aged animals (Weible et al., 2004), and supports the role of cholinergic agonists in rescuing working memory performance in AD and aged human subjects (Hampel et al., 2018). In addition, the observation that trace conditioning is learned efficiently when a specific brain state such as the theta rhythm period, is chosen in old animals (Asaka et al., 2005), might also indicate an increase in the endogenous levels of acetylcholine that might enhance the learning of the task. Finally, our observation that aged CA1 neurons can support intact persistent firing suggests that this cellular mechanism may be one of several mechanisms activated when cognitive performance is rescued by cholinergic activation. Therefore, specific activation of this mechanism could be a candidate drug target for therapeutic treatment which could be more specific than cholinergic activation (Lu et al., 2017).

## Synthesis

Reviewing Editor: Susanne Wolf, Charite University Hospital Decisions are customarily a result of the Reviewing Editor and the peer reviewers coming together and discussing their recommendations until a consensus is reached. When revisions are invited, a fact-based synthesis statement explaining their decision and outlining what is needed to prepare a revision will be listed below. The following reviewer(s) agreed to reveal their identity: Rachel Anderson.

1. In terms of methods and result descriptions, it is difficult to understand how behavioral training is related to the experiment design.

2. in vitro brain slice experiments, which must undergo acute brain slices, hypoxia or injury, young mice are more tolerated than older mice, and it is difficult to determine that changes in these parameters are not caused by different results from acute injury? And the quality of slice between aged mice (24 M) and young one should be really different, such as survived neurons, network......How to determine the change in capacitance is not a matter of brain slice quality, for example, survived neurons are more likely to edema in early and old mice.

3. It should be noted that the agonist induced persistent firing is far away from event-locked high-frequency discharge in vivo studies.

3. results, the input resistance decreased in aged group, and the excitability decreased, but the threshold did not change, how to explain? This maybe why the carbachol (agonist) induced persistent firing did not changes in aged animal? There are changes in AHP, looks like K+ related mechanism is more important than Ach. in aged group mice? A large part of the effect of choline on the hippocampus is at the threshold of neuronal discharge, so the absence of significant changes in agonist-induced sustained firing here may be related to the constant threshold, or it may be a problem of excessive agonist concentration.

4.In Figure 4, traces of Figure A does not match well with the input resistance of the previous result, and the initial discharge frequency may differ significantly between groups in the first 2 seconds when bin=2 seconds. And DB should explained more clear in panel, like which drug was used to block, time course...

5. need explain “carbachol” when it be first used.

## Author Response

Letter to the editor and reviewers

We very much thank the editor and reviewers for the constructive comments and suggestions to enhance the quality of our manuscript. Please find below a point-by-point reply addressing the reviewers’ concerns. We hope we addressed all comments adequately. Comments from the reviewers are in italics and underlined for clarity. All changes in the revised manuscript are shown in red.

Synthesis Statement for Author:

1. In terms of methods and result descriptions, it is difficult to understand how behavioral training is related to the experiment design.

We thank the reviewer for raising this point. Behavioral training was not related and did not influence experimental design. We report it here solely for the purpose of describing the nature of the aged group rats used in our study. We think it is important to do so based on previous publications reporting the effect of behavioral training on cellular excitability in a different strain of rat (Kuo et al. 2008, Oh, Oliveira, and Disterhoft 2010) and persistent firing in a different strain and type of cell (Lin et al. 2020). Following this comment, we added a sentence in the method and result sections to make this point clearer as follows:

Methods section: “This behavioral training was not part of our experimental design. We report it here solely for the purpose of describing the nature of aged group rats used in our study based on previous publications reporting the effect of behavioral training on cellular excitability in a different strain of rat (Kuo et al. 2008, Oh, Oliveira, and Disterhoft 2010) and persistent firing in a different strain and type of cell (Lin et al. 2020). “

Results section: “This behavioral training was not part of our experiment design.”

2. in vitro brain slice experiments, which must undergo acute brain slices, hypoxia or injury, young mice are more tolerated than older mice, and it is difficult to determine that changes in these parameters are not caused by different results from acute injury? And the quality of slice between aged mice (24 M) and young one should be really different, such as survived neurons, network......How to determine the change in capacitance is not a matter of brain slice quality, for example, survived neurons are more likely to edema in early and old mice.

We thank the reviewers for raising this critical matter. As mentioned, the slice preparation quality tends to degrade with the animal’s age and this may have an impact on the excitability of cells. In fact, it has been reported that slice preparation conditions can significantly alter cellular excitability in juvenile and aged animals more than in adults (Lauderdale, 2015).

While it still remains technically challenging to fully prevent edema and damage in acute brain slices, some approaches we adopted helped to curb the loss of quality. For instance, to reduce cell depolarization following inevitable anoxia during slice preparation steps, we have used a choline-based cutting solution for all age groups (see method). This has been reported to reduce the cellular permeability of sodium channels during slice preparation and protect cell morphologies in CA1 even at very advanced ages (Moyer and Brown, 1998; Ting et al., 2014).

Additionally, the health of slice preparation and cells vary not only depending on the age but also day to day, and cell to cell. Therefore, we used criteria such as the resting membrane potential, spike height, and stability to selectively include only healthy cells. These criteria were applied equally to all age groups to ensure similar levels of health and stability across groups.

Finally, it should be noted that cellular properties have historically been compared between different age groups using similar in vitro methods to our current study in a number of studies (Disterhoft and Oh 2007, Lin et al. 2020, Matthews, Linardakis, and Disterhoft 2009, Spigelman et al., 1992). Overall, our results fit with most of the reported tendencies in juvenile and aged CA1 cells from Long-Evans rats (Severin et al., 2020).

3. It should be noted that the agonist induced persistent firing is far away from event-locked high-frequency discharge in vivo studies.

We appreciate the reviewers’ concerns and agree that agonist-induced intrinsic persistent firing is different from persistent firing observed in event-locked discharge in vivo. The presented agonist-induced persistent firing is proposed as a potential cellular mechanism underlying working memory-related persistent firing. We have added a sentence to the discussion to address this point:

Discussion section: “Motivated by the potential role of persistent firing as a cellular mechanism underlying working memory functions and cholinergically mediated age-dependent decline of cognitive performance, in this paper, we compared persistent firing in young rats (14-24 days) with old rats (24-27 months) in an in vitro preparation”.

4. results, the input resistance decreased in aged group, and the excitability decreased, but the threshold did not change, how to explain? This maybe why the carbachol (agonist) induced persistent firing did not changes in aged animal? There are changes in AHP, looks like K+ related mechanism is more important than Ach. in aged group mice? A large part of the effect of choline on the hippocampus is at the threshold of neuronal discharge, so the absence of significant changes in agonist-induced sustained firing here may be related to the constant threshold, or it may be a problem of excessive agonist concentration.

In our manuscript, we showed that lower input resistance in the aged group caused lower excitability. However, the spike threshold was intact, probably because input resistance does not contribute to the spike threshold (Yamada-Hanff and Bean, 2013). Input resistance reflects the size of the cell and the number of ion channels open during the measurement. The channels influencing the resistance in these measurements should be those which are open at rest such as sodium and potassium leak channels (e.g., NALCN and the K2P family, respectively). The spike threshold, on the other hand, depends mainly on voltage-gated sodium channels (e.g., Nav1). Our observation suggests that input resistance and excitability probably decreased due to the larger size of the cells in the aged group as reported in Vasilyev & Barish, 2002, without affecting channels that contribute to the spike threshold. As stated in the original manuscript (p18, line 391-393), the carbachol-induced current was larger in aged animals, which we suggest may have counteracted the decreased excitability during persistent firing. However, as pointed out by the reviewer, in the hippocampus acetylcholine exerts a large effect on currents such as the M-current (KCNQ/Kv7 channels) that is activated at around spike threshold. Therefore, if the firing threshold were higher in aged rats, then this increased current might not have been sufficient to cause a similar level of persistent firing.

Regarding the relative importance of K+-related mechanisms over the acetylcholine-related mechanisms, it is difficult to conclude whether these K+-related mechanisms had a larger role in the aged group compared to the young group. As a matter of fact, in the aged group, mAHP was smaller while sAHP was increased (Fig.4D-H). In addition, K+ channels that underlie mAHP and sAHP are modulated by acetylcholine (Cole and Nicoll, 1983). Therefore, it is also difficult to separate K+ mechanisms from acetylcholine. If the reviewer is wondering whether K+ channel suppression is sufficient to induce persistent firing, it has been reported that blocking the M-current (one of the K+ channels) is not sufficient to support persistent firing in CA1 pyramidal cells in young rats (Knauer and Yoshida, 2019). We don’t know, however, if this is also the case in aged rats.

Finally, we cannot exclude the possibility that the absence of difference in persistent firing from young and aged rats in our study could be due to the relatively high level of agonist we used. This is now mentioned in the discussion section as follows:

Discussion section: “It should, however, be pointed out that we cannot exclude the possibility that the relatively high concentration of carbachol we used in our study could mask a hypothetical difference in persistent firing between the young and aged groups at a lower level of cholinergic activation.”

5.In Figure 4, traces of Figure A does not match well with the input resistance of the previous result, and the initial discharge frequency may differ significantly between groups in the first 2 seconds when bin=2 seconds. And DB should explained more clear in panel, like which drug was used to block, time course... We thank the reviewers for the suggestions. Traces in Figure A are meant to focus on similar persistent firing frequencies across ages. Therefore, we show traces used to measure persistent firing, where the cells are held at a membrane potential just below the threshold, as opposed to ones where input resistance was measured from a -70 mV baseline. In addition, the Ca2+-dependant CAN current that rapidly initiates during the 2-second step stimulation is stronger in aged rats, which may be the reason why we can’t detect a difference in excitability between young and aged rats during the stimulation to induce persistent firing. The trace in figure 4.A showing the depolarization block (DB) has been modified following the reviewers’ comments: the part of the trace showing DB has been marked with a bracket. The drug used during the recording is now mentioned for each trace (ACSF+Cch). Following the reviewers’ suggestions, we ran additional analyses on the persistent firing frequencies.

However, we were not able to see any significant difference in the initial frequency with bin = 2 seconds (RM Two-Way Anova: p-value = 0.685, F(3,31) = 0.500).

6. need explain “carbachol” when it be first used. We apologize for the insufficient explanation. Carbachol is now defined at the first occurrence in the result section as “a non-selective cholinergic agonist”.

Best regards,

The authors (names are omitted for anonymous review process)
